# Pericardial malignant infiltration as the cause of sudden death of a patient with metastatic urothelial carcinoma treated with atezolizumab

**DOI:** 10.1186/s12894-022-01064-x

**Published:** 2022-07-18

**Authors:** Patrik Palacka, Pavol Janega, Hana Polakova, Jan Slopovsky, Valentina De Angelis, Michal Mego

**Affiliations:** 1 2nd Department of Oncology, Faculty of Medicine, Comenius University in Bratislava, Bratislava, Slovakia; 2 National Cancer Institute, Klenova 1, 833 10 Bratislava, Slovakia; 3 Department of Pathology, Faculty of Medicine, Comenius University in Bratislava, Bratislava, Slovakia; 4 Department of Radiology, National Cancer Institute, Bratislava, Slovakia

**Keywords:** Metastatic urothelial carcinoma, Atezolizumab, Complete response, Sudden death, Malignant pericardial infiltration

## Abstract

**Background:**

Muscle-infiltrating urothelial carcinoma of the bladder is the most common genitourinary cancer. Immunotherapeutic agents targeting protein-1 programmed death or protein-1 programmed death ligand are currently considered the standard treatment in patients with either inoperable locally advanced or metastatic urothelial carcinoma (MUC) after platinum-based chemotherapy failure.

**Case presentation:**

Here we report the case of a Caucasian male patient with metastatic urothelial carcinoma treated with second-line atezolizumab within a trial who achieved complete response by computed tomography (CT), but suddenly died due to cardiac tamponade resulting from malignant pericardial infiltration. Histopathology confirmed this as the only site of disease progression.

**Conclusions:**

Cardiovascular toxicity of atezolizumab was considered within differential diagnoses, however histopathological examination revealed progression of malignancy in the pericardium as the cause of the sudden death. This is the first published case report of a patient treated with second-line atezolizumab in whom the rare disease progression of pericardial infiltration was confirmed. Despite its rarity, the clinicians should always consider the possibility of pericardial metastases.

## Background

Bladder carcinoma is the ninth most common malignancy with an overall incidence of 20.4 cases per 100,000 population. The most common bladder cancer is urothelial carcinoma, which accounts for 90% of all malignant bladder tumours [1]. The most common sites of metastases are extra loco-regional lymph nodes, bones, lungs, liver, and peritoneum. Pericardial metastases, while quite rare, present a risk of sudden cardiac death [2]. In this case report, we present a 66-year-old patient with metastatic urothelial carcinoma (MUC) treated with atezolizumab as second-line within the phase 3 SAUL study (NCT02928406).

Despite computed tomography (CT) showing a complete response, the patient died suddenly as a result of cardiac tamponade caused by metastases to the pericardium, the only site of disease progression that could be identified.

To the best of our knowledge this is the first case report of isolated pericardial metastases in a patient with metastatic urothelial carcinoma (MUC) treated with immunotherapy based on protein-1 programmed death (PD-1) or PD-L1 (protein-1 programmed death ligand) inhibition.

## Case presentation

A 66-year-old Caucasian male underwent right-sided uretero-nephrectomy due to high-grade muscle-infiltrating urothelial carcinoma, pT2N0M0, stage II in October 2006 and radical cystectomy with Mainz pouch II urinary diversion for high-grade muscle-infiltrating urothelial bladder carcinoma, pT2N0M0, stage II in April 2010. The malignant urothelial cells from bladder were positive for cytokeratin CK7 and cytokeratin CK20, and showed negativity for p63, vimentin and calretinin. The patient was observed by a urologist.

On March 3, 2017, an enlarged lymph node in the right groin was surgically removed and relapse of urothelial carcinoma was confirmed by histopathology examination. Abdominal, pelvic and thoracic computed tomography (apt-CT) with contrast was performed on March 27, 2017. This showed lymphadenopathy (LAP) in the left supraclavicular area, in the retroperitoneum, bilaterally in the para-iliac area, and in both groins. Bajorin prognostic score (performance status > 1 and the presence of visceral involvement) was zero in this patient. Median survival of patients with no negative prognostic factors was 33.0 months in this study by Bajorin et al. [3].

From March 28, 2017 to July 18, 2017, this patient underwent six courses of gemcitabine (G, 2000 mg/m^2^ intravenously on days 1 and 8) with cisplatin (C, 70 mg/m^2^ intravenously on day 1) without any serious adverse event of grades 3 and 4. Only grade 1, anemia and fatigue were recorded. The first apt-CT scan on May 31, 2017, showed a complete response, which was confirmed by subsequent apt-CT scan after GC completion on August 10, 2017. Then, the patient was observed by an oncologist. Cardiac function was assessed by echocardiography before GC initiation. Only type 1 diastolic dysfunction of the left ventricle (LV) and a slight degenerative rearrangement of the valves were determined. LV ejection fraction was 63%, no pericardial effusion was present.

On November 10, 2017, pelvic magnetic resonance imaging (MRI) revealed localized pathological LAP close to the left external iliac vessels and in the left groin area, clinical scrotal oedema was present. Due to localized relapse, external beam radiotherapy (ExRT) to the total dose of 40 Grays (Gy) was applied to the area of LAP from November 13, 2017 to December 12, 2017 without any serious acute toxicity.

**Fig. 1:**
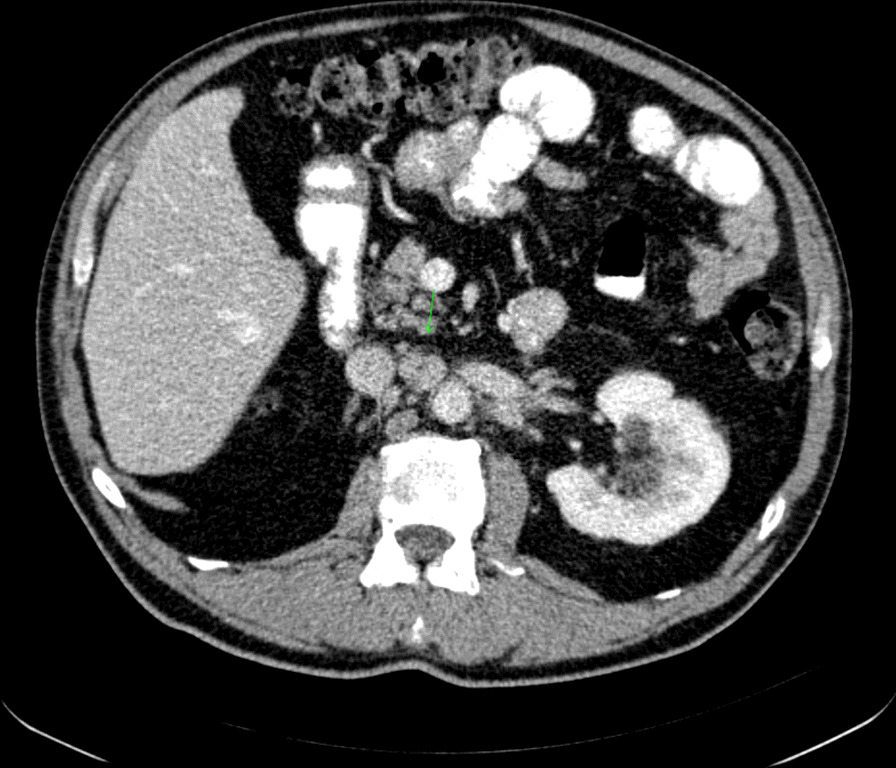
Computed tomography scan before atezolizumab treatment initiation (January 17, 2018): aorto-caval retroperitoneal lymph node—the target lesion (25 mm × 18 mm)—green arrow

A restaging apt-CT scan was performed on January 17, 2018, revealing an enlarged aortocaval retroperitoneal lymph node sized 25 mm × 18 mm (a target lesion, Fig. 1) and another enlarged retroperitoneal lymph node sized 39 mm × 13 mm (a non-target lesion). Bellmunt prognostic score (performance status < 0, hemoglobin level less than 10 g/dL, and the presence of liver metastasis) was zero in this patient. Median survival of patients without any negative prognostic factors was 14.2 months in this Bellmunt study [4].

**Fig. 2:**
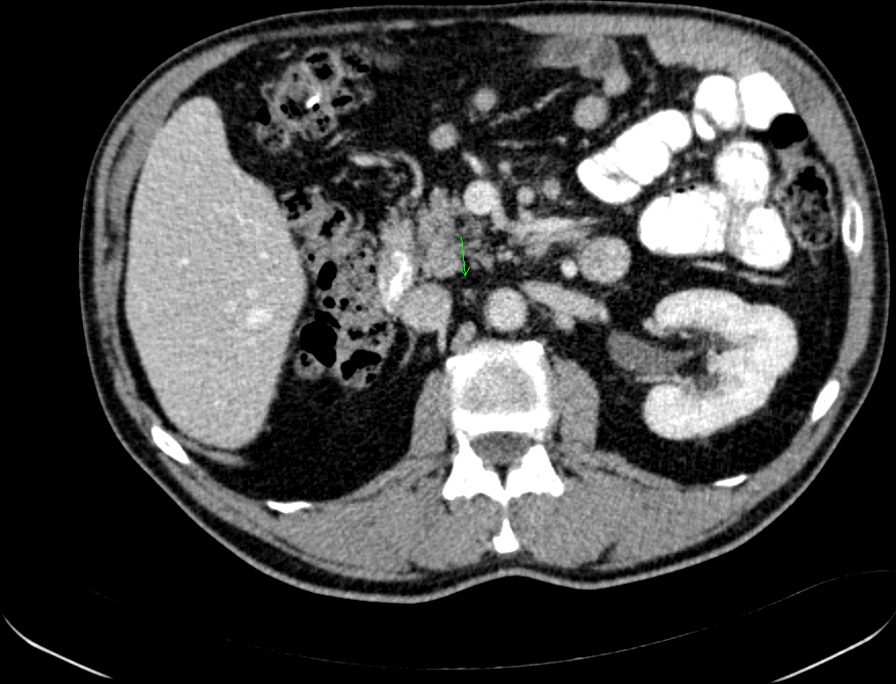
Computed tomography scan at the time of pre-planned restaging (August 9, 2018): Aortocaval retroperitoneal lymph node—the target lesion (6 mm × 5 mm)—green arrow

From January 17, 2018, this patient who met the inclusion criteria was treated within the clinical trial NCT02928406 (ClinicalTrials.gov Identifier) with atezolizumab (1200 mg intravenously every 3 weeks), a monoclonal antibody against PD-L1. On the pre-planned restaging apt-CT scan on August 8, 2018, complete remission was shown. The enlarged aortocaval retroperitoneal lymph node (a target lesion) decreased in size from 25 mm × 18 mm to 6 mm × 5 mm (Fig. 2); however clinical scrotal oedema persisted. The patient was treated with immunotherapy until September 20, 2018 without any serious (grades 3 and 4) adverse event. Only grade 1 fatigue was recorded during this systemic therapy.

On October 4, 2018, the patient was admitted to the hospital with clinical deterioration in terms of anxiety and restlessness, weakness, rapid breathing, fainting, dizziness followed by rapid loss of consciousness. On admission, hypotension (blood pressure 60/35 mm Hg), peripheral cyanosis, and distant heartbeat sounds on auscultation were present. Increase in aminotransferases (alanine aminotransferase 6300 IU/L, aspartate aminotransferase 6300 IU/L), creatine of 3.62 mg/dL, hyponatremia, hyperkalemia, hypo-coagulation state, and metabolic acidosis were also recorded. Electrocardiogram (ECG) showed low QRS voltage and sinus tachycardia (120 per minute). Echocardiography could not be utilized because during physical examination, the patient went into pulmonary circulatory arrest, resuscitation was unsuccessful and he died due to cardiorespiratory failure.

**Fig. 3:**
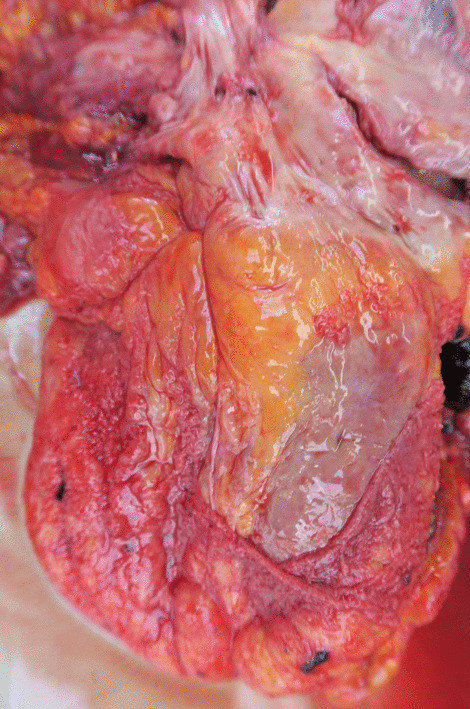
The complete pericardium covered by gray-to-white coatings and infiltrated by neoplastic cells, histologically corresponding to the metastatic dissemination of muscle-infiltrating urothelial carcinoma

**Fig. 4:**
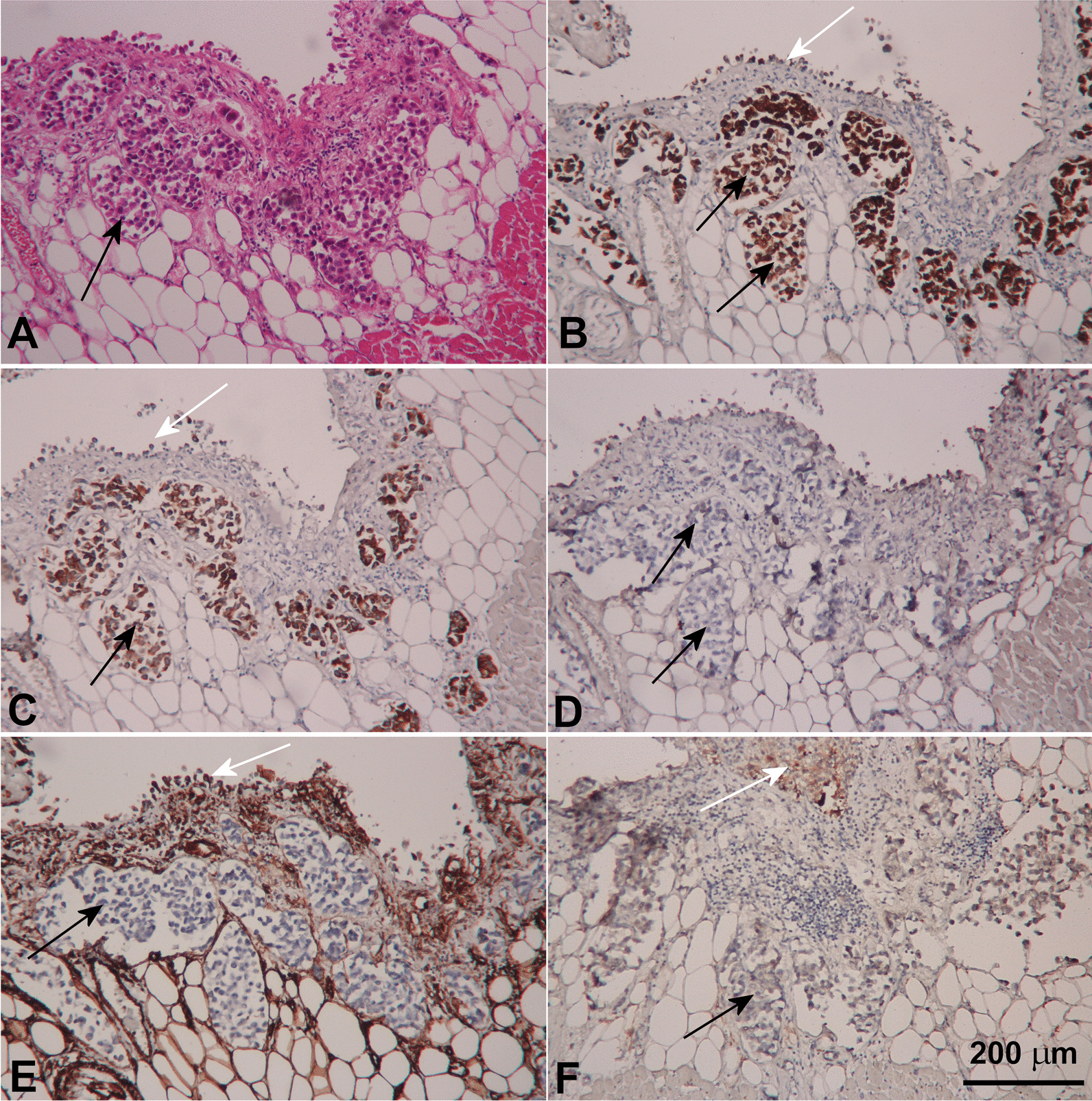
Histological examination showed pericardial infiltration by high grade urothelial carcinoma (**A**). The neoplastic cells (black arrows) showed positivity for cytokeratin markers CK7 (**B**) and CK20 (**C**) and were negative for p63 (**D**), vimentin (**E**) and calretinin (**F**), when compared to the activated mesothelial cells (white arrows) weakly positive for CK7 (**B**) and negative for CK20 (**C**) with regular positivity for vimentin (**E**) and calretinin (**F**)

**Fig. 5:**
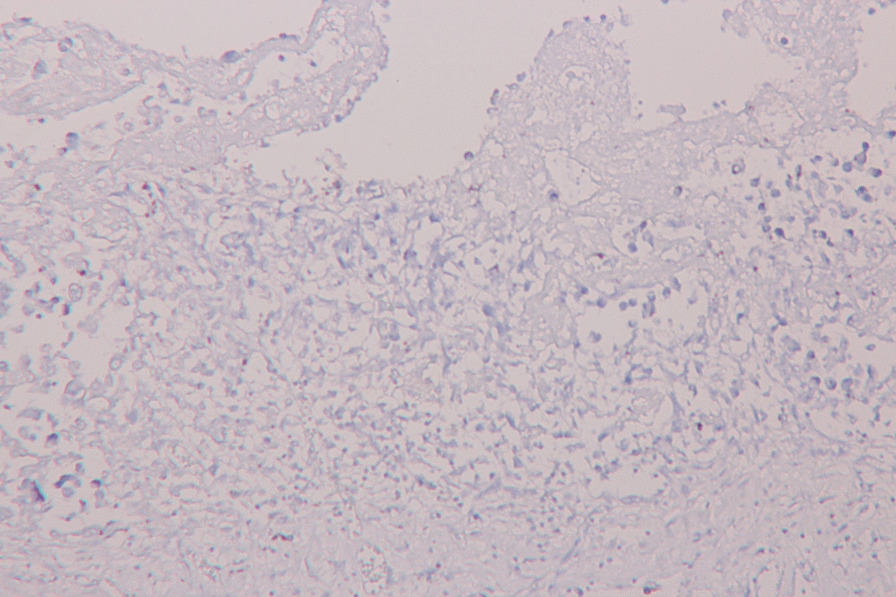
Negative PD-L1 status determined immunohistochemically using the rabbit monoclonal antibody against PD-L1 (28-8 clone, Merck Cell Marque, Rocklin, California, USA)

In making differential diagnoses, we considered the toxicity of atezolizumab since complete response was present on the most recent CT re-evaluation. On pathological examination, no macroscopic visible neoplasia was found. However, in the pericardial cavity a small amount (less than 50 ml) of sanguinolent effusion was present and the pericardium was covered by irregular grey coatings. The complete pericardium was covered by gray-to-white coatings and in evaluated sections infiltrated by neoplastic cells, histologically corresponding to the metastatic dissemination of invasive urothelial carcinoma (Fig. 3). Microscopic examination (Fig. 4) showed thickening of the pericardium infiltrated by neoplastic proliferation and the formation of irregular nests of epithelioid cells with marked pleomorphism, enlarged hyperchromatic nuclei with a high n/c ratio. No significant inflammatory infiltration was present. Immunohistochemical examination confirmed pericardial infiltration by high grade urothelial carcinoma. The neoplastic cells showed positivity for cytokeratin markers CK7 and CK20 and were negative for p63, vimentin and calretinin. The activated mesothelial cells were weakly positive for CK7, negative for CK20 and p63 and showed regular positivity for vimentin and calretinin. PD-L1 expression was analyzed immunohistochemically using the rabbit monoclonal antibody against PD-L1 (28–8 clone, Merck Cell Marque, Rocklin, California, USA). PD-L1 status was negative (Fig. 5). The paraaortic lymph nodes were not significantly enlarged, with elastic consistence and gray color on cross-section. No metastatic dissemination was found in evaluated in lymph nodes.

In this patient, progression-free survival (PFS) following first-line GC treatment was 8.3 months and 8.5 months for second-line immunotherapy. Overall survival (OS) from initiation of systemic therapy until death was 19.1 months.

## Discussion and conclusions

The pericardium is the most common site of cardiac metastasis (64–69%) followed by the epicardium (25–34%) and the myocardium (29–32%) [5]. However, the possibility of pericardial metastases in patients with metastatic urothelial carcinoma is not widely recognised. In autopsies of 17 of patients with MUC, the rate of metastasis to the epicardium was approximately 11% [6]. These metastases present an increased risk of cardiac tamponade, malignant arrhythmias and subsequent death. Initial presentation of cardiac metastasis may vary, from asymptomatic to various heart rhythm abnormalities such as atrial fibrillation, atrioventricular blocks or ventricular fibrillation, or signs of cardiac tamponade [5]. Physical examination during anticancer therapies plays an important role, because suspicion of pericardial disease can be raised through findings such as decreased heart sounds, new onset cardiac murmur, development of pericardial friction rub, hypotension with narrow pulse pressure and distended jugular veins [7]. ECG evaluation can be of value but often shows nonspecific ST-T deformities [8]. On echocardiography, pericardial effusions or cardiac masses can sometimes be seen [9]. Treatment is based on urgent pericardiocentesis despite its high recurrence rates (30–60%) [10]. In patients with a longer life expectancy, surgical drainage, based on effusion location and clinical condition, offers the lowest recurrence rate [10].

PD-1 is expressed by activated T lymphocytes. It attaches with its ligands PD-L1 to restrict the activation of T lymphocytes. Monoclonal antibodies targeting PD-1 and PD-L1 can block the immune checkpoint and eliminate the inhibition of T lymphocytes activation. As a result, they enhance immune reactions to fight against malignancies. However, due to its blockade of inhibitory regulation of T-lymphocytes and therefore unregulated activation of immune reaction, it may also affect other organs and injure normal tissues, leading to immune-related adverse effects [11].

In a single arm, phase 2 study [12] with atezolizumab, side effects of any grade were present in 69% of subjects and majority of them were mild to moderate in nature. The most common toxicities of atezolizumab were fatigue, nausea, decreased appetite, and pruritus. However, in 16% of cases, the adverse events (mostly fatigue) were serious. The immune-mediated side effects of any grade were present in 7% of patients, with pneumonitis, increased aspartate aminotransferase and alanine aminotransferase being the most common. 5% of subjects had a serious immune-mediated adverse event. There were no treatment-related deaths reported in this study.

In IMvigor-211 trial [13], in both the expression on ≥ 5% of tumor-infiltrating immune cells (IC2/3) and intention-to-treat (ITT) populations, grade 3 or 4 treatment-related adverse events were less common with atezolizumab than chemotherapy. Among the 467 patients treated with atezolizumab (ITT population), treatment-related adverse events were reported as follow: fatigue (2%), anaemia (2%), neutropenia (< 1%), peripheral neuropathy (< 1%), asthenia (2%), and febrile neutropenia (< 1%). In both trials with atezolizumab the rate of pericarditis and pericardial effusion were not reported. So far there are limited data about the rate of occurrence of pericardial disease in patients treated with ICIs.

In our patient treated with atezolizumab, histopathological examination confirmed the urothelial immunophenotype (CK7 +, CK20 +) of the neoplastic cells within the pericardium. Impaired p63 expression, observed in this case, was found in other studies to be associated with higher biological aggression of high-grade urothelial carcinoma [14–16]. When we searched through PubMed using the key words “pericardial infiltration”, “urothelial carcinoma”, “high grade”, and “atezolizumab” no articles were found. Therefore, we believe that this is the first case report of a patient with metastatic urothelial carcinoma who achieved complete CT response with atezolizumab after the failure of cisplatin-based chemotherapy, but who suddenly died due to malignant tumour infiltration of the pericardium as the only site of disease progression.

In this patient, OS calculated from atezolizumab initiation was shorter (8.5 months) than expected survival (14.2 months) considering the Bellmunt prognostic score. However, the Bellmunt study [4] identified pretreatment prognostic factors for OS only in MUC patients who experienced treatment failure with the first-line, platinum-based regimen included in the phase 3 vinflunine trial. In the phase 2 and phase 3 studies [12, 13] with atezolizumab, median OS was 11.4 months and 11.1 months, respectively.

In conclusion, immunotherapy based on check-point inhibition, regardless of PD-L1 expression, is the current standard treatment for patients with advanced urothelial carcinoma after failure of platinum-based combined chemotherapy. In comparison to cytostatic treatment, immunotherapy improves both survival and the percentage of objective responses including complete responses while having fewer adverse effects in general.

Any new treatment carries the risk of new side effects, which may be causally related to the systemic therapy. However, the sudden death of our patient was not related to atezolizumab, but was due to malignant pericardial infiltration confirmed by histopathological examination, despite the complete response to immunotherapy on CT. It is therefore important that the possibility of pericardial metastases should not be discounted, especially where any cardiopulmonary symptoms are evident.

## Data Availability

The data and materials are available from the corresponding author.

## Abbreviations

apt-CT: Abdominal, pelvic, and thoracic–computed tomography
ECG: Electrocardiogram
ExRT: External radiotherapy
GC: Gemcitabine plus cisplatin
ITT: Intention to treat
LAP: Lymphadenopathy
MUC: Metastatic urothelial carcinoma
MRI: Magnetic resonance imaging
OS: Overall survival
PFS: Progression-free survival
PD-1: Programmed cell death protein–1
PD-L1: Programmed death protein–1 ligand
